# A real-world study on unmet medical needs in triptan-treated migraine: prevalence, preventive therapies and triptan use modification from a large Italian population along two years

**DOI:** 10.1186/s10194-019-1027-7

**Published:** 2019-06-27

**Authors:** Carlo Piccinni, Sabina Cevoli, Giulia Ronconi, Letizia Dondi, Silvia Calabria, Antonella Pedrini, Immacolata Esposito, Valentina Favoni, Giulia Pierangeli, Pietro Cortelli, Nello Martini

**Affiliations:** 1 Fondazione ReS (Ricerca e Salute) – Research and Health Foundation, Via Magnanelli 6/3, Casalecchio di Reno, 40033 Bologna, Italy; 2 IRCCS Istituto delle Scienze Neurologiche di Bologna, Bologna, Italy; 3 Drugs and Health, Rome, Italy; 4 Department of Biomedical and Neuromotor Sciences, University of Bologna, Bologna, Italy

**Keywords:** Real-world evidence, Burden of disease, Italy, Administrative databases, Observational study, Pharmacoepidemiology

## Abstract

**Background:**

Although migraine is a disabling neurological condition that causes important disability, it remains an area of underdiagnosis and undertreatment worldwide. The aim of this study was to depict the burden of the unmet medical needs in migraine treated with triptans in a large Italian population.

**Methods:**

A 2-year longitudinal analysis of migraineurs with unmet medical needs on treatment with triptans was performed. The studied cohort consisted of subjects with ≥4 triptan dose units per month, selected from the general population These patients were stratified into: possible Low-Frequency Episodic Migraine (pLF-EM: 4–9 triptan dose units per month), possible High-Frequency Episodic Migraine (pHF-EM: 10–14 triptan dose units per month) and possible Chronic Migraine (pCM:> 14 triptan dose units per month). The first follow-up year was analysed to describe the use of preventive therapies, the second year to describe the ≥50% reduction in triptan use.

**Results:**

Of 10,270,683 adults, 8.0 per 1000 were triptan users and, of these, 38.2% were migraineurs with unmet medical needs, corresponding to 3.1 per 1000 adults. By stratifying for the number of triptan dose units per month, 72.3% were affected by pLF-EM, 17.4% by pHF-EM, and 10.3% by pCM. In this cohort, 19.1% of individuals used oral preventive drugs and 0.1% botulinum toxin. Triptan use reduction was found in 22.3% individuals of the cohort, decreasing with the intensification of need levels (25.8% pLF-EM, 13.6% pHF-EM, 12.0% pCM).

**Conclusions:**

This real-life analysis underlined that the unmet medical needs concern a large part of patients treated with triptans and there is an undertreatment with preventive therapies whose benefit is insufficient, which may be due to the lack of effective preventive strategies, probably still reserved to severe patients. This study allows forecasting the actual impact of newest therapeutic strategies aimed to fill this gap.

## Introduction

The World Health Organization has declared migraine the third most common disease and the second cause of disability [1]. It affects roughly 12–14% of the adults in occidental countries and it is most common in the age class 35–45 years old, with a prevalence three times higher in women than in men [2, 3]. In Europe, the estimate of migraine prevalence comes mainly from studies based on personal interviews or questionnaires: a mean prevalence of 14.7% has been found, with few Italian studies included in this estimate [4]. This elevated prevalence is also associated with a huge economic impact, both as direct and indirect costs: a study estimated that for migraine the mean per-person annual costs were €1222 with a total annual cost of €111 billion for 27 European Countries [5].

The International Classification of Headache Disorders 3rd edition defined chronic migraine (CM) as “headache occurring on 15 or more days/month for more than three months, which, on at least eight days/month, has the features of migraine headache” [6]. Consequently, episodic migraine (EM) refers to patients having headache for 14 or less days per month. CM has an estimated worldwide prevalence of 1.4% to 2.2% [7], and the rate of EM evolving into CM is 2.5% per year [8]. In Italy, there are scarce epidemiological data on CM: a questionnaire-based study involving 16,577 subjects reported that the prevalence of people suffering from headache attacks for more than 15 days per month was 3.4%, despite many patients being misdiagnosed by general practitioners [9]. Furthermore, CM is associated with higher medical resource use and total costs compared to EM, therefore the use of treatments aimed to reduce the number of attacks could decrease the clinical and economic burden of this disease [10].

Although migraine is a considerable clinical problem, it remains an area of significant unmet medical needs and of underdiagnosis and undertreatment worldwide [11] as well as in Italy [12].

To date, pharmacological care of migraine headaches includes acute therapies aimed to relieve the symptoms during the attacks and preventive therapies that should decrease the attack frequency in order to improve responsiveness to acute therapies. Beside these, several non-pharmacological approaches, such as nutraceuticals, behavioural techniques and acupuncture, are gaining ground in the clinical management of migraine [13].

Acute therapy of migraine attacks consists of triptans, ergot derivatives, and analgesics (NSAIDs) to which an antiemetic can be added [14]. Out of these, only triptans and ergot derivatives are specifically labelled for migraine attacks treatment. Different oral drugs, originally developed for other indications such as epilepsy, depression or high blood pressure, can be used to prevent migraine attacks [15–17]. Finally, in 2013 the injection of botulinum toxin A was approved as preventive therapy in CM patients non-responders to oral preventive therapies [18, 19]. Finally, recently monoclonal antibodies directed against the calcitonin gene-related peptide have been introduced among therapeutic strategies for migraine with the promise to become the new frontier of treatment for this disease [20, 21].

In this scenario, it has become essential to know the real-life burden of unmet medical needs (UMN) in migraine, by distinguishing between migraine with “high” medical needs and migraine with “low” medical needs. Another important piece of information to obtain concerns the use of available preventive therapies and the estimate of their effectiveness in real practice. This can be achieved by using real-world data collected into health administrative databases and analysed with the strategies of the real-world evidence [22, 23].

The aim of this study was, therefore, to describe the prevalence and characterisation of unmet medical needs in migraine among triptan users by distinguishing between high (possible CM) and low (possible EM) needs. Moreover, the study has provided a real-world landscape of the use of headache preventive therapies and of an estimate of their effectiveness in terms of reduction in triptan use.

## Methods

### Data source

A real-world analysis was performed by using ReS DataBase (ReS DB), coming from the collaboration between ReS (Fondazione Ricerca e Salute – Research and Health Foundation) and CINECA (Interuniversity Consortium). ReS DB is a patient-centred data warehouse that includes the following healthcare administrative databases linked with each other: socio-demographic registry, reimbursed drug prescriptions database, hospital discharges database, and outpatient services and visits database.

ReS DB, for the period 2012–2015, collected information of more than 12 million of Italian inhabitants resident in different Regions and Local Health Units. Quality and usefulness of this data source is guaranteed by several studies published in the international literature [24, 25].

### Study design and patient selection

This is a longitudinal analysis of triptan-treated migraine patients selected from a representative sample of the general Italian population.

Patients affected by migraine were identified among subjects aged ≥18 years and receiving at least one triptan prescription from January 1, 2013 to December 31, 2013, by searching in the pharmaceutical database the ATC (Anatomical Therapeutic Chemical) code N02CC (Selective serotonin - 5HT1 agonists). The first triptan prescription date represented the index date for each subject. The observational periods included 24 months post-index date (follow-up period, until December 31st, 2015). Only patients with available data for the entire period (2013–2015) were included in the analysis.

Starting from the index date, the 1-year triptan prescription history was analysed to compute the monthly average number of dose units collected by each patient. Because the prescription database collects information on drug dispensing and not on its actual intake, to mitigate possible discrepancies between these two aspects, for each subject the sum of dose units dispensed in 1 year was divided by 12 months to obtain the monthly average number of collected dose units. This value could be considered similar to the actual number of dose unit intakes per patient. By taking a triptan dose unit as a proxy of a day with migraine attack, a subject was considered affected by unmet medical needs (UMN) in migraine if he/she received an average of 4 or more triptan dose units per month in 1 year. This threshold was established analogously to several clinical trials for migraine prophylaxis setting “4 days of migraine per month” as the lower limit for entering into the studies.

The cohort of UMN migraineurs treated with triptan, according to the average number of triptan dose units per month, was stratified into three groups: possible Low-Frequency Episodic Migraine (pLF-EM; from 4 to 9 triptan dose units per month); possible High-Frequency Episodic Migraine (pHF-EM; from 10 to 14 triptan dose units per month); possible Chronic Migraine (pCM; more than 14 triptan dose units per month).

### Prescription patterns of preventive therapies

To describe the complete prescription pattern of triptan-treated migraineurs, in the 12 months post-index date the presence of prescriptions of the following oral preventive therapies reimbursed by the Italian National Health System (NHS) was searched: topiramate (ATC: N03AX11), valproic acid (N03AG01), propranolol (C07AA05), metoprolol (C07AB02), atenolol (C07AB03), timolol (C07AA06), amitriptyline (N06AA09) and pizotifen (N02CX01). Moreover, in the same period the use of botulinum toxin was searched and identified by the presence of specific drug dispensation (ATC: M03AX01) or by the presence of the specialist outpatient procedure of injection (procedure code: 99.29.9 – injection of botulinum toxin).

### Assessment of triptan use reduction

This analysis was based on the assumption that a notable reduction in the mean number of triptan intakes could be a reliable proxy of migraine improvement. For each patient the prescription history of triptans was analysed (with the same strategy described above) in two different periods: from the index date to the 12th month and from the 13th month to the 24th month. By comparing these two periods, a patient was defined “improved” if his/her mean number of triptan dose units per month decreased by at least 50%.

### Statistical analysis

The prevalence estimates of overall triptan users and, among these, of UMN migraineurs (overall and for different subgroups) were provided per 1000 adult inhabitants. UMN migraineurs treated with triptan were described in terms of demographic characteristics (gender and age). Moreover, all preventive therapies (oral drugs and botulinum toxin injections) used in the year pre-index date were described as rate of treated subjects, number of different drugs, specific active substance. Finally, the rates of improved subjects were provided for the overall UMN triptan-treated migraine cohort and for each subgroup. Moreover, the improvement rates were analysed in presence and absence of oral preventive therapies or botulinum toxin injection. A chi-square test was performed to compare the frequencies of improvement between subjects with or without preventive therapy (oral drugs or botulinum toxin injection). A difference was considered statistically significant when the *p* value was <.01. All statistical analyses were performed by Oracle SQL Developer ver. 17.

## Results

### Prevalence and socio-demographic characteristics of UMN migraineurs

Starting from a population of 12,217,384 subjects resident in 6 Italian Regions in 2013, 10,270,683 were adults (aged 18+ years old) and, out of these, 85,048 (0.83% of adults) received at least one prescription of triptan during 2013. To conduct the study 82,446 triptan users with data available for the entire observational period were selected (i.e. excluding patients who died or moved to Regions not included in ReS DB). Therefore, the prevalence of triptan use was of 8.0 per 1000 adult inhabitants.

**Fig. 1:**
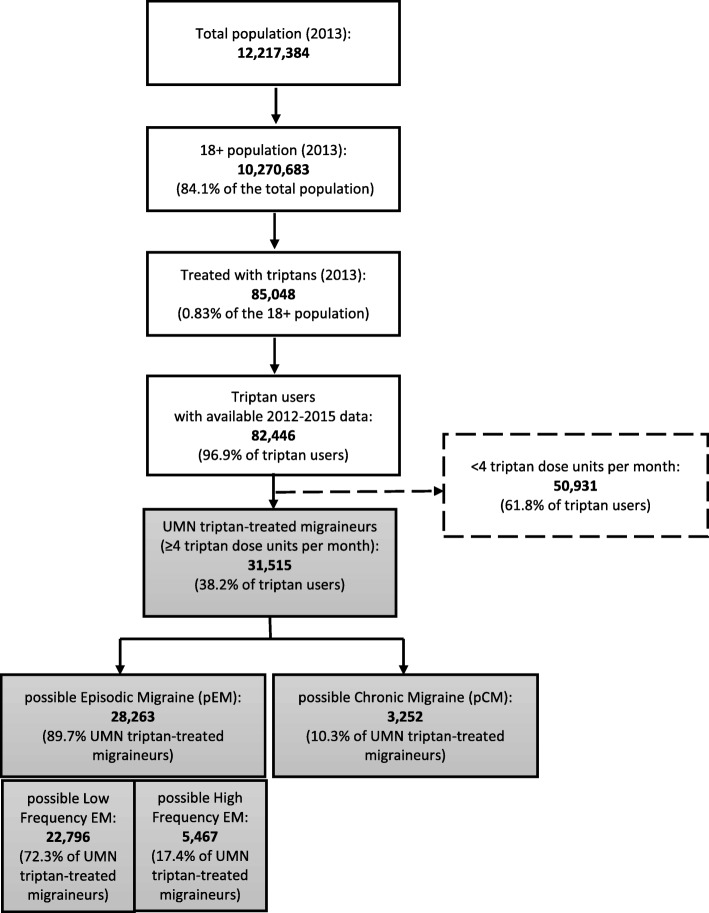
Selection of unmet medical needs (UMN) in triptan-treated migraineurs among triptan users and their distribution into different migraine subtypes according to the average triptan dose units per month

Among these triptan users, 31,515 patients turned out to be affected by UMN in migraine (i.e. with 4 or more triptan dose units per month) with a prevalence of 3.1 per 1000 adult inhabitants. By stratifying this cohort according to the average triptan dose units per month, 28,263 proved affected by pEM (89.7% of UMN migraineurs) and 3252 by pCM (10.3%). Among pEM patients, 22,796 (72.3% of UMN migraineurs) were affected by pLF-EM and 5467 (17.4%) by pHF-EM. Therefore, the prevalence estimates of different UMN triptan-treated migraine subtypes were: 2.2 per 1000 for pLF-M, 0.5 for pHF-EM and 0.3 for pCM (Fig. 1 and Table 1).

**Table 1:** Prevalence and demographic characteristics of triptan users and unmet medical needs (UMN) in migraineurs treated with triptans, for the overall cohort and stratified for subtypes according to the average triptan dose units per month

	Overall triptan users	Overall UMN migraineurs	Average triptan dose units per month in 1 year		
			4–9	10–14	> 14
			pLF-EM	pHF-EM	pCM
	82,446	31,515	22,796	5467	3252
Rate of triptan users (%)	100	38.2	27.6	6.6	3.9
Rate of UMN migraineurs (%)	–	100	72.3	17.4	10.3
Prevalence (per 1000 adult inhabitants)	8.0	3.1	2.2	0.5	0.3
Gender					
Female (%)	78.5	80.9	81.5	81.0	76.2
Male (%)	21.5	19.1	18.4	19.0	23.8
Age					
Mean ± SD	47 ± 13	49 ± 11	48 ± 11	49 ± 11	50 ± 11
Median	47	48	48	48	49
18–29 (%)	9.8	5.0	5.5	3.6	4.0
30–39 (%)	18.0	14.4	15.8	14.0	12.1
40–49 (%)	32.0	36.2	33.0	37.1	35.4
50–59 (%)	24.1	28.6	27.7	29.5	29.8
60–69 (%)	10.9	11.7	13.5	11.5	13.0
70–79 (%)	3.9	3.5	3.9	3.5	4.8
≥ 80 (%)	1.2	0.7	0.6	1.0	0.8

*UMN* Unmet medical need, *pLF-EM* Possible low frequency episodic migraine, *pHF-EM* Possible high frequency episodic migraine, *pCM* Possible chronic migraine

Among triptan users, female gender was higher than male (78.5% F vs. 21.5% M) and this difference was more pronounced among UMN migraineurs treated with triptan (80.9% vs. 19.1%). This gender imbalance was found in all UMN triptan-treated migraine subtypes, although it was slightly lower for pCM (76.2% F vs. 23.8% M).

The mean age of triptan users was 47 years old and it was higher for those subjects affected by UMN migraine and treated with triptan (49 years old). The mean age of UMN triptan-treated migraineurs increased according to the mean number of triptan dose units per month: from 48 years old for pLF-EM to 50 years old for pCM. The distribution of subjects for the different age groups, in all studied groups, showed a percentage increase until the age group 40–49 years old, and a decrease in the subsequent age groups. Finally, 5.1% of triptan users, and 4.7% of UMN triptan-treated migraineurs, were ≥ 70 years old, although triptans are not recommended in elderly subjects (Table 1).

### Preventive therapies

Out of all UMN migraineurs on triptan treatment, during the year post-index date 21.3% received at least one oral drug as headache preventive therapy. This percentage increased according to the triptan dose units received per month: it was 18.8% among patients with pLF-EM, 25.2% among those with pHF-EM, and it reached the peak of 32.8% among patients with pCM. Patients treated with oral preventive therapy received a single active substance, in percentages around 80% in all different subgroups. Nevertheless, the percentage of subjects with prescriptions of two or more different oral preventive drugs increased according to the augmentation of triptan dose intake.

By analysing the specific active substances, amitriptyline was the most prescribed drug (9.4% of the cohort), followed by topiramate (6.3%), propranolol (3.3%) and atenolol (2.7%). The same ranking was found for all subgroups.

The injection of botulinum toxin in the year post-index date occurred in 0.3% of UMN triptan-treated migraineurs; this rate increased with the augmentation of the average triptan dose units used by patients: from 0.2% of pLF-EM to 1.0% of pCM (Table 2).

**Table 2:** Use of migraine preventive therapies (oral drugs and botulinum toxin) among triptan-treated migraineurs with unmet medical needs (UMN) (overall cohort and stratified for the average triptan dose units per month) in the first year of follow-up

	Overall UMN migraineurs	Average triptan dose units per month in 1 year		
		4–9	10–14	> 14
		pLF-EM	pHF-EM	pCM
	31,515	22,2796	5467	3252
Oral Preventive Therapies in the 1st year				
Treated with at least one drug (%)	21.3	18.8	25.2	32.8
1 drug (% of treated subjects)	82.9	84.9	82.0	75.7
2 drugs (% of treated subjects)	15.1	13.8	15.4	20.2
3 drugs (% of treated subjects)	1.8	1.1	2.4	3.8
4 drugs (% of treated subjects)	0.2	0,2	0,2	0,3
Active substance:				
Amitriptyline (%)	9.4	8.5	11.1	13.0
Topiramate (%)	6.3	5.0	8.5	12.0
Propranolol (%)	3.3	2.8	4.1	5.3
Atenolol (%)	2.7	2.6	2.5	4.1
Valproic acid (%)	1.8	1.4	2.0	3.9
Pizotifen (%)	1.0	0.8	1.2	2.4
Metoprolol (%)	0.6	0.6	0.7	1.1
Timolol (%)	0.3	0.3	0.3	0.3
Botulinum Toxin in the 1st year				
Treated (%)	0.3	0.2	0.5	1.0

*UMN* Unmet medical need, *pLF-EM* Possible low frequency episodic migraine, *pHF-EM* Possible high frequency episodic migraine, *pCM* Possible chronic migraine

### Triptan use reduction

A triptan use reduction, as proxy of migraine improvement, was estimated by comparing the mean number of triptan dose units per month in two subsequent years. In the overall cohort of UMN migraineurs receiving triptans, 22.3% of patients improved (i.e. with a ≥ 50% reduction of triptan dose units per month). The rate of improvement decreased with the intensification of triptan intake, ranging from 25.8% for pLF-EM to 12.0% for pCM.

The improvement rate was significantly lower among subjects treated with oral preventive therapies in comparison with those without these drugs (19.7% vs. 22.9%, *p* < .01). The same analysis, performed in different subgroups, showed that among pLF-EM patients those treated with oral preventive therapies had a lower frequency of improvement than those not treated with these therapies (23.7% vs 26.2%; *p* < .01). On the contrary, among pCM patients, the improvement rate of subjects with oral preventive therapies was significantly higher than of those not treated with these drugs (14.5% vs. 10.9%; *p* < .01). Also the patients treated with botulinum toxin injection showed a lower improvement rate compared to those without this treatment, however this difference was not statistically significant among overall UMN migraineurs (15.7% vs. 22.3%; *p* = .03) (Table 3).

**Table 3:** One-year migraine improvement, in terms of modification in triptan use, among triptan-treated migraineurs with unmet medical needs (UMN) (both overall cohort and stratified for subtypes according to the average triptan dose units per month)

	Total	Not Improved		Improved^a^		*P* value^
	N	N	(%)	N	(%)	
Overall UMN migraineurs (≥ 4 triptan dose units)	31,515	24,503	(77.7)	7012	(22.3)	
At least one oral preventive therapy in the 1st year						
Yes	6121	4913	(80.3)	1208	(19.7)	<.01
No	25,394	19,590	(77.1)	5804	(22.9)	
At least one botulinum toxin injection in the 1st year						
Yes	191	161	(84.3)	30	(15.7)	.03
No	31,324	24,342	(77.7)	6982	(22.3)	
pLF-EM (4–9 triptan dose units)	22,796	16,916	(74.2)	5880	(25.8)	
At least one oral preventive therapy in the 1st year						
Yes	3856	2944	(76.3)	912	(23.7)	<.01
No	18,940	13,972	(73.8)	4968	(26.2)	
At least one botulinum toxin injection in the 1st year						
Yes	71	46	(64.8)	25	(35.2)	.07
No	22,725	16,870	(74.2)	5855	(25.8)	
pHF-EM (10–14 triptan dose units)	5467	4725	(86.4)	742	(13.6)	
At least one oral preventive therapy in the 1st year						
Yes	1278	1125	(88.0)	153	(12.0)	.06
No	4189	3600	(85.9)	589	(14.1)	
At least one botulinum toxin injection in the 1st year						
Yes	49	48	(98.0)	1	(2.0)	
No	5418	4677	(86.3)	741	(13.7)	.02
pCM (> 14 triptan dose units)	3252	2862	(88.0)	390	(12.0)	
At least one oral preventive therapy in the 1st year						
Yes	987	844	(85.5)	143	(14.5)	<.01
No	2265	2018	(89.1)	247	(10.9)	
At least one botulinum toxin injection in the 1st year						
Yes	71	67	(94.4)	4	(5.6)	.10
No	3181	2795	(87.9)	386	(12.1)	

*UMN* Unmet medical need, *pLF-EM* Possible low frequency episodic migraine, *pHF-EM* Possible high frequency episodic migraine, *pCM* Possible chronic migraine

^ The differences of frequencies of migraine improvement between subjects with or without preventive therapy were tested through a chi-square test, considering statistically significant a *p* value <.01

^a^Improved defined as reduction of ≥50% in average triptan dose units per month by comparing two subsequent years

## Discussion

This study, based on real-world data collected into healthcare administrative databases, provides the actual burden, among triptan users, of the unmet medical needs (UMN) in migraine in Italy, distinguishing for different need levels. The study reported that 8.0 per 1000 adults were on treatment with triptan, although the prevalence of migraine is estimated to be 12–14% of the general population [2, 3].

Moreover, the analysis showed that the prevalence of UMN migraine treated with triptans was 3.1 per 1000 adults. This finding, although in the awareness that it is lower than the estimates reported by other studies based on different methodologies, should be evaluated in relation to the overall prevalence of triptan use. Therefore, on the basis of this result it is possible to state that a large part of triptan users could be considered unmet medical need migraineurs (3.1 of 8.3 per 1000). Thus, these findings reveal that in the group of migraineurs receiving triptans (a therapy generally subsequent to a correct diagnosis by neurologists) there is still a large part of migraineurs without sufficient relief of their condition. Moreover, the showed distribution of different need levels should be taken into account to estimate the rate of migraine sufferers who can benefit more from new therapies.

In our study, we used the prescription of triptan as proxy of migraine diagnosis and we adopted the average monthly dose unit as proxy of headache attacks. A similar strategy was used by another Italian study [26] based on electronic health records of general practitioners and obtaining results comparable to ours. Moreover, to date, several studies used healthcare databases to investigate the use of triptans, both concerning Italian settings [27, 28] and other countries, such as France, the Netherlands, Germany and Australia [29–32]. Nevertheless, no epidemiological data were derived from these data sources on the real-life UMN in migraine, which represents an important issue in the neurological field. Although we are aware that some migraineurs could be not treated with triptans, both due to clinical reasons (e.g. intolerance) and to a missed treatment without reasons, these drugs are specifically indicated for the acute treatment of migraine attacks and they are reimbursed by the Italian NHS (with routine recording into the Outpatient Drug Prescription database). Indeed, triptan prescriptions can be considered a reliable proxy of migraine disease when administrative databases are analysed.

Additional important results of this study concern the actual use of preventive therapies and the rate of migraine improvement among triptan-treated subjects. As a matter of fact, the oral preventive therapies and botulinum injection were used in a low percentage of UMN migraineurs treated with triptans. This percentage was low also without excluding subjects on treatment with such drugs (e.g. beta-blockers) for a specific comorbidity (e.g. cardiovascular disease), since the preventive effect of a drug on headache attacks should be independent of the presence/absence of a comorbidity. This aspect was even more relevant among subjects with CM who generally suffer more from comorbidities [33]. The very low use of all available preventive therapies could reflect a scarce benefit of these strategies and it underlines an unmet clinical need, especially for those subjects treated with > 14 triptan dose units per month, which are potentially affected by CM. This was confirmed by the findings on migraine improvement, defined in this study as a reduction in triptan use, which occurred only in a low percentage of UMN migraineurs receiving triptans, with decreasing rates as the triptan treatment intensified. The improvement rate was lower among subjects treated with oral preventive drugs compared with those not treated with these drugs, especially among patients with low need level (i.e. pLF-EM subjects). On the contrary, a significantly higher improvement rate was observed only among subjects with high need level (i.e. pCM patients) treated with oral preventative drugs in comparison with those not treated with these therapies. No significant differences were found in improvement rates in presence or absence of botulinum toxin injection. However, these last results were affected by some drawbacks: the very low number of patients receiving this treatment and the inability to identify the actual number of attacks at the botulinum initiation, especially among CM subjects for whom the botulinum treatment is indicated. These preliminary results point out that a large part of subjects suffering from migraine, especially those with high medical needs, to date do not receive an effective treatment, with consequences for their health, quality of life and economic burden for the entire system, as also argued by previous studies [10, 12].

## Strengths and limitation

The main strengths of this study were the magnitude of the studied population and the possibility of analysing all triptan prescriptions as the Italian NHS reimburses these drugs. Therefore, this study was able to provide a reliable picture of the real management of triptan-treated migraine. On the other hand, the inability to identify the entire population affected by migraine (i.e. patients not treated with triptans or patients that purchased them out of pocket), represented the main limitation of our analysis. Although also non-treated migraineurs could be UMN patients, this study, by using administrative data, demonstrated that even among triptan users there was a significant proportion of UMN patients. Another drawback concerns the analysis of preventive therapies: in our study we included only drugs labelled for migraine prevention and reimbursed by the Italian NHS. This is due to the fact that administrative databases only record reimbursed drugs and not out-of-pocket ones. Therefore, this study did not take into account other preventive strategies such as not-reimbursed drugs (e.g. flunarizine), as well as non-pharmacological treatments, such as nutraceuticals or behavioural techniques and acupuncture [13]. We are also aware that in EU countries the approval of botulinum toxin A as preventive therapy in CM patients occurred in the first quarter of 2013, therefore our analysis, performed on the 2013–2015 period was able to describe only the initial use of botulinum toxin for migraine.

An additional strength of this study, based on real-world data, is the provision of the actual panorama on drug use for migraine acute and preventive treatment. However, this analysis was affected by the well-known drawbacks of research studies based on administrative databases [34], in particular, the possible discrepancy between drug dispensation and actual drug intake, or the lack of information on precise indication and other clinical features that could drive the drug choice.

Finally, concerning migraine improvement, since we used as proxy the reduction (≥50%) in the mean number of triptan doses, our findings should be considered as preliminary outcomes and they should be confirmed or rejected by future studies based on different methodologies or by integrating different types of data, as suggested by other researchers [35, 36]. This also considering that this last analysis could be affected by misclassification since some subjects stopping the triptan treatment and considered “improved” actually could be intolerant or resistant to these drugs, which is a condition involving several patients [37].

## Conclusion

In conclusion, this study showed that in the Italian real practice there is a large part of migraineurs with unmet medical needs also among those patients treated with triptan. This aspect should be taken into account in the health organisation for appropriate management of migraine. Indeed, not all migraine patients can attend third-level headache centres, but some sort of “stepped care” beginning at the primary (general practitioners) and secondary care levels (general neurological ambulatories) could meet the needs of a certain portion of the patients with UMN.

Moreover, the study underlined that the current use of preventive therapies is scarce and with negligible benefits. This could be due to the lack of effective strategies to reduce the number of headache attacks. In view of the new drugs being introduced for the prevention of migraine [21], this study could represent a model to design future studies aimed to analyse appropriateness in the management of migraine and to help policy decision makers in the resource allocation for this disease. Furthermore, the results of this study could be helpful in the setting of critical pathways for migraine aimed to improve patient outcomes and reduce the direct and indirect costs of this clinical condition.

## Data Availability

The datasets generated and analysed during the current study are not publicly available, they come from anonymised administrative databases owned by the Italian Regional Health Facilities and they were analysed by Fondazione ReS under a specific agreement.

## Abbreviations

ATC: Anatomical Therapeutic Chemical
CM: Chronic migraine
EM: Episodic migraine
NHS: National Health System
NSAIDs: Non Steroidal Anti Inflammatory Drugs
pCM: Possible Chronic Migraine
pHF-EM: Possible High Frequency Episodic Migraine
pLF-EM: Possible Low Frequency Episodic Migraine
ReS DB: Ricerca e Salute DataBase
UMN: Unmet Medical Need
